# The complexity of patients hospitalized in Internal Medicine wards evaluated by FADOI-COMPLIMED score(s). A hypothetical approach

**DOI:** 10.1371/journal.pone.0195805

**Published:** 2018-04-16

**Authors:** Erminio Bonizzoni, Gualberto Gussoni, Giancarlo Agnelli, Raffaele Antonelli Incalzi, Moira Bonfanti, Franco Mastroianni, Marco Candela, Carlotta Franchi, Stefania Frasson, Antonio Greco, Micaela La Regina, Roberta Re, Giorgio Vescovo, Mauro Campanini

**Affiliations:** 1 Department of Clinical Science and Community, Section of Medical Statistics, Biometry and Epidemiology, Faculty of Medicine and Surgery, University of Milan, Milan, Italy; 2 Research Department FADOI Foundation, Milan, Italy; 3 Internal Medicine and Cardiovascular-Stroke Unit, S. Maria della Misericordia Hospital, University of Perugia, Perugia, Italy; 4 Gerontology Unit, University Hospital Campus Bio-Medico, Rome, Italy; 5 Internal Medicine, San Giuseppe Hospital, Empoli, Italy; 6 Internal Medicine, F. Miulli Hospital, Acquaviva delle Fonti, Bari, Italy; 7 Internal Medicine, E.Profili Hospital, Fabriano, Ancona, Italy; 8 Laboratory for Quality Assessment of Geriatric Therapies and Services, Department of Neuroscience, IRCCS- Istituto di Ricerche Farmacologiche Mario Negri, Milan, Italy; 9 Internal medicine, Hospital Casa Sollievo della Sofferenza, San Giovanni Rotondo, Italy; 10 Internal Medicine, Hospital of La Spezia, La Spezia, Italy; 11 Department of Internal Medicine, Hospital ‘Maggiore della Carita’, Novara, Italy; 12 Internal Medicine, Sant’Antonio Hospital, Padova, Italy; Universita degli Studi di Napoli Federico II, ITALY

## Abstract

**Objectives:**

The aim of this study is to develop a new predictive model to measure complexity of patients in medical wards.

**Setting:**

29 Internal Medicine departments in Italy.

**Materials and methods:**

The study cohort was made of 541 consecutive patients hospitalized for any cause, aged more than 40 years and with at least two chronic diseases. First, we applied a hierarchical cluster analysis and the principal component analysis (PCA) to a panel of questionnaires [comorbidity (Charlson, CIRS), clinical stability (MEWS), social frailty (Flugelman), cognitive dysfunction (SPSMQ), depression (5-item GDS), functional dependence (ADL, IADL, Barthel), risk of sore threats (Exton-Smith scale), nutrition (MNA), pain (NRPS), adherence to therapy (Morisky scale)], in order to select domains informative for the definition of complexity. The following step was to create the score(s) needed to quantify it.

**Results:**

Two main clusters were identified: the first includes 7 questionnaires whose common denominator is dependence and frailty, the second consists of 3 questionnaires representative of comorbidity. Globally, they account for about 70% of the total variance (55.2% and 13.8%, respectively). The first principal component was simplified in “Complimed Score 1” (CS1) as a recalibrated average between the Barthel Index and the Exton Smith score, whereas the second cluster was approximated to “Complimed Score 2” (CS2), by using the Charlson score only.

**Conclusions:**

Complexity is a two-dimensional clinical phenomenon. The FADOI-Complimed Score(s) is a new tool useful for the routine evaluation of complexity in medical patients, simple to use and taking around 10 minutes to complete.

## Introduction

The majority of patients hospitalized in general medical wards are elderly, with multiple and usually chronic diseases. Age and multimorbidity (including illness severity and interrelatedness among diseases, functional status of the patient and the need for multiple treatments) are determinants of clinical outcome. However, they do not completely define the complexity of medical patients, which is also affected by a number of additional elements, the most relevant being the functional dependence of the patient; cognitive disorders and psychological attitudes; familiar, socioeconomic and environmental status; and the organization of healthcare [1–4].

Defining and measuring patients’ complexity is a difficult task [5,6], but may have significant implications for prognosis, clinical decision-making, organization of care and allocation of resources. A number of validated non-disease specific prognostic indices for older adults have been proposed, but in many cases, they have limitations both on methodological bases and real-life utilization [7]. Indeed, several indices require the collection of information that is rarely assessed in routine clinical practice, and is time-consuming, impeding their widespread use and application. Among the available tools, the Multidimensional Prognostic Index (MPI) has provided a reliable method of stratifying 1-year mortality risk in elderly patients [8], and its use has increased in recent years. However, it is possible that the MPI itself does not comprehensively measure the complexity of patients; further, despite it being less complicated than other indices, it still includes 63 assessments to be carried out and this limits its applicability in daily clinical practice.

In this paper, we propose a new tool used in the setting of Internal Medicine (IM) departments, which is aimed at assessing the complexity of hospitalized patients and helping the physician in prognostic stratification. For the development of this tool we employed a two-stage strategy which is summarized as follows:

- in the first stage, we applied a hierarchical cluster analysis to a panel of representative questionnaires in order to bring out the domains potentially involved in complexity, and to obtain a selection by eliminating those deemed insufficiently informative;
- in the second stage, a principal component analysis was performed in order to better characterize the findings of cluster analysis and to develop the scores needed to quantify the degree of complexity.

## Materials and methods

### Study procedures

Consecutive patients admitted for any cause to 29 IM Units in Italy during the period June-October 2014 were eligible, if aged over 40, and suffering from at least two chronic diseases on hospital admission.

**Fig 1:**
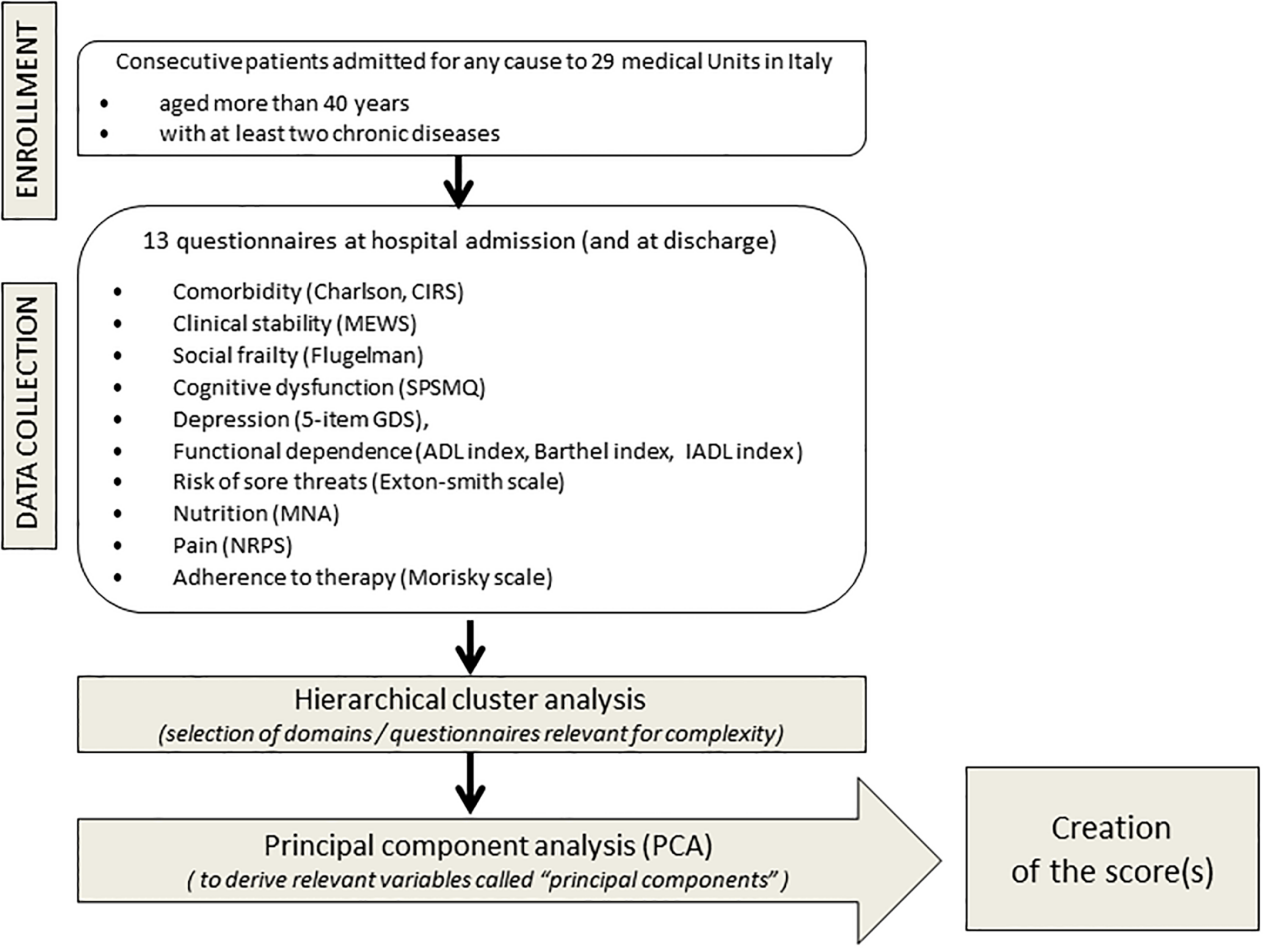
Flow-chart of the methods used for the study.

The methods used for the study and described in detail below, are summarized with a graphical representation in Fig 1. Patient general information (demography, routine laboratory tests, social environment/support, diseases, drug therapy) was collected on admission. Thirteen questionnaires [9–21] were administered to each patient to evaluate domains considered representative of the complexity of patients: comorbidity (Charlson, CIRS), clinical stability (MEWS), social frailty (Flugelman), cognitive dysfunction (SPSMQ), depression (5-item GDS), functional dependence (activities for daily living: ADL Katz index and Barthel index; instrumental activities for daily living: IADL), risk of developing sores (Exton-Smith scale), nutrition (MNA), pain (NRPS), and adherence to therapy (Morisky scale). In case the collection of information scheduled by the protocol was not feasible (due to critical clinical conditions of the patient and/or severe cognitive dysfunction), study procedures could be delayed during hospital stay until clinical conditions improved, or information could be collected with the help of relatives or caregivers. For the purposes of the study, help from relatives/caregivers in completing questionnaires was needed in 14% of the patients enrolled. All the information collected through the questionnaires referred to the time of hospital admission, apart from IADL, whose items applied to one week prior to hospitalization, and Flugelman, ADL and Barthel, which were considered for both hospital admission and one week before. A sensitivity analysis was also performed by considering the same set of information collected at the time of hospital discharge. While choosing the panel of questionnaires, the Scientific Committee of the study evaluated those available for each domain, and selected those already validated and more frequently used in clinical practice. As an additional criterion, the questionnaires and information to be considered for the MPI score were included, with a view to comparing the new tool we were creating with MPI as a reference. Further, we tried to be as careful as possible, in order to avoid neglecting important information, even if this could lead to the inclusion of variables only tenuously linked to complexity. Specific training was provided to researchers in order to standardize and optimize the administration of the questionnaires, the completion of the clinical record form and data collection.

The study hypothesis was that the degree of complexity would be quantifiable through one or more derived variables obtained from the information shared by most of the questionnaires. It follows that the first step of the analysis should be aimed at identifying the domains potentially involved in complexity, while allowing the selection of only those which were likely to contain relevant information, and this could be accomplished by using hierarchical clustering algorithms.

Through cluster analysis, questionnaires are arranged into natural groupings with sizes that should reflect the clinical importance of the associated complexity domains. The relationship between cluster size and the relevance of the domain is inherent to the composition of the panel of questionnaires, since it is expected that: “the larger the number of panel questionnaires related to a specific trait of complexity, the greater the weight given to the trait itself”.

Hence, the empirical evidence suggesting that the clinical importance of a complexity domain depends on the size of the related cluster supports the conclusion that unclustered questionnaires (namely the cluster formed by one questionnaire only) should represent the complexity domains that are least relevant, and therefore, could potentially be removed from the subsequent analyses with a minimal loss of information.

After eliminating unclustered questionnaires, the principal component analysis was used to extract the joint information of remaining questionnaires and convert it into one or more derived variables called “principal components”. The number and composition of the retained principal components allowed us to establish both the dimension of complexity, and to interpret the nature of the information contained therein.

Once the dimension and nature of complexity was ascertained, the next step was to create the scores needed to quantify it. These scores had to be simple and ideally obtained only from one or a few original questionnaires, while adhering as strictly as possible to the information provided by the principal components.

### Ethics statement

The study was conducted according to the Good Clinical Practice Guidelines and the Declaration of Helsinki. According to Italian law, a preliminary approval for the study was obtained by the Ethics Committee of the coordinating center—Comitato Etico (CE) Milano Area C—A.O. Niguarda Ca’ Granda, Milan, and after that all the Ethics Committees of the participating centers gave their approval (CE Provinciale di Crotone, CE Regionale del Friuli VG, CE Ospedale "San Donato" di Arezzo, CE Regionale delle Marche, CE dell’IRCCS "Casa Sollievo della Sofferenza" di S. Giovanni Rotondo (FG), CE ASL Roma G, CE di Legnano, CE dell’AO Cardarelli di Napoli, CE dell’Ospedale Maggiore della Carità di Novara, CE "S. Croce e Carle" di Cuneo, CE dell’Ospedale "Bianchi-Melacrino-Morelli" di Reggio Calabria, CE Provinciale di Vicenza, CE Provinciale di La Spezia, CE dell’Ospedale "S. Camillo Forlanini" di Roma, CE della Provincia di Agrigento, CE dell’AUSL di Bologna, CE dell’Ospedale "Miulli" di Acquaviva nelle Fonti, CE "ARNAS Garibaldi" di Catania, CE dell’Ospedale "Mauriziano" di Torino, CE Provinciale di Savona, CE Regionale dell’Umbria, CE dell’AOU "Careggi" di Firenze, CE dell’Azienda Ospedaliera dei Colli di Napoli).

### Informed consent

Written informed consent was obtained from all participating patients. In case of patients unable to give their informed consent, due to severe physical or psychological/ cognitive conditions, was the legal representative responsible for signing the consent. The need to resort to the signature by the legal representative was left to the judgment of the attending physician, and linked to the patient’s ability to respond adequately to questions of a general nature and related to the state of health.

### Statistical methods

The sample size in this study satisfies both the “rule of thumb” of 20–30 times as many cases as parameters, and the “absolute minimum sample size” rule which states that a sample larger than 400 is highly recommended in a principal component analysis [22].

Cluster analysis was carried out using the clustering algorithm implemented in the procedure VARCLUS of SAS. The VARCLUS algorithm is a type of oblique component analysis which is particularly useful as a method of variable reduction. It is used to divide a set of numeric variables into disjoint or hierarchical clusters, which can be graphically displayed through a dendrogram. In our analysis, we used a proportion of variance and chose around 80% as a reasonable cut-off point for clustering the questionnaires.

Principal component analysis (PCA) was carried out using the procedure PRINCOMP of SAS. PCA is a multivariate technique usually used to reduce the dimensionality (number of variables) of a large number of interrelated variables, while retaining as much of the information (variation) as possible. PCA calculates an uncorrelated set of derived variables (principal components), which are ordered so that the first few retain most of the variation present in all of the original variables. The computations of PCA reduce to an eigenvalue-eigenvector problem. In fact, each principal component is a linear combination of the original variables, with coefficients equal to the eigenvectors of the correlation matrix. The principal components are sorted in descending order of the eigenvalues, which are equal to the variances of the principal components. Because the sum of all eigenvalues corresponds to the number of the original variables, we dropped those principal components whose eigenvalues were below one, since these provide less information than is provided by a single variable (Kaiser criterion). In addition, if the designated number of principal components did not account for at least 50% of the variance, then the whole analysis was aborted and our initial assumptions reconsidered. Finally, concurrent validity was assessed by comparing PCA findings with available validated tools by means of robust linear regression analysis (Passing-Bablok Median-Slope Regression) and proper correlation indices (Kendall’s Tau Correlation Coefficient and Lin’s Concordance Correlation Coefficient). For the purposes of cluster and principal component analysis, the questionnaires ADL, IADL, Barthel, Exton-Smith, Morisky and MNA were recalibrated so that they ranged from the lowest (best) to the highest (worst). All computations were performed using SAS software version 9.4.

## Results and discussion

### General characteristics of patients, and results of the questionnaires

A total of 541 patients were enrolled in the study. The general characteristics of the study population, and results of the questionnaires are detailed in Table 1. As expected, the study population was made up of elderly patients with multiple diseases and undergoing concomitant treatments, and with a not negligible grade of social frailty and functional dependence.

**Table 1:** Baseline characteristics of patients (n = 541). Values are expressed as mean ± standard deviation or percentages.

**Age (years)**	78.2 ± 9.8
< 65 years	10.1%
65–74 years	19.5%
75–84 years	43.4%
≥ 85 years	27.0%
**Female**	51%
**BMI**	25.7 ± 5.6
**Chronic diseases**	
Heart failure	35.6%
Chronic obstructive pulmonary disease	35.6%
Diabetes	33.0%
Moderate/severe renal insufficiency	28.4%
Cancer	18.4%
Moderate/severe liver insufficiency	9.4%
**Number of drugs**	
at home	6.1 ± 3.4
on admission to Internal Medicine	6.4 ± 3.7
**Caregiver YES**	67.4%
**Questionnaires / scales**	
ADL	2.4 ± 2.5
Barthel	53.9 ± 39.3
Charlson	4.0 ± 2.6
CIRS	3.4 ± 1.8
Exton-Smith	14.8 ± 4.3
Flugelman	12.1 ± 4.0
GDS	2.0 ± 1.6
IADL	4.0 ± 3.0
MEWS	1.3 ± 1.2
MNA	18.6 ± 5.8
Morisky	3.2 ± 1.2
NRPS	2.2 ± 3.0
SPMSQ	3.1 ± 3.4

### Hierarchical cluster analysis

**Fig 2:**
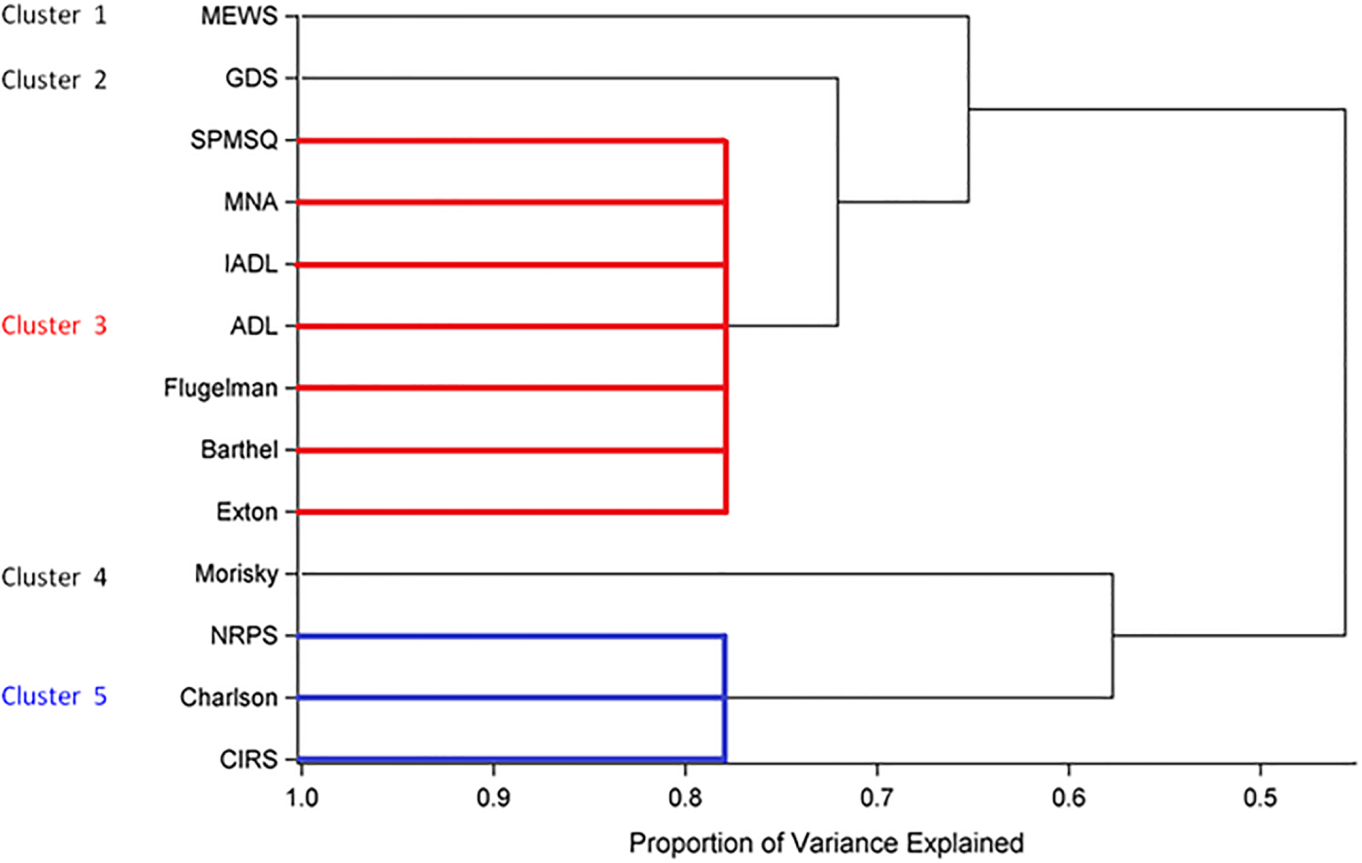
Dendogram of the cluster analysis.

The dendogram reported in Fig 2 shows that the thirteen questionnaires segregate into five distinct clusters with three of them formed by one element only. The number and composition of the retrieved clusters is consistent with one of the scenarios of complexity hypothesized during the phase of definition of the panel of questionnaires. Based on these results and in line with study procedure, the unclustered questionnaires MEWS, GDS and Morisky were judged insufficiently informative and therefore were not included in the principal component analysis. The sensitivity analysis performed by using data collected at discharge led to the same conclusions.

### Principal component analysis

#### Interpretation

**Fig 3:**
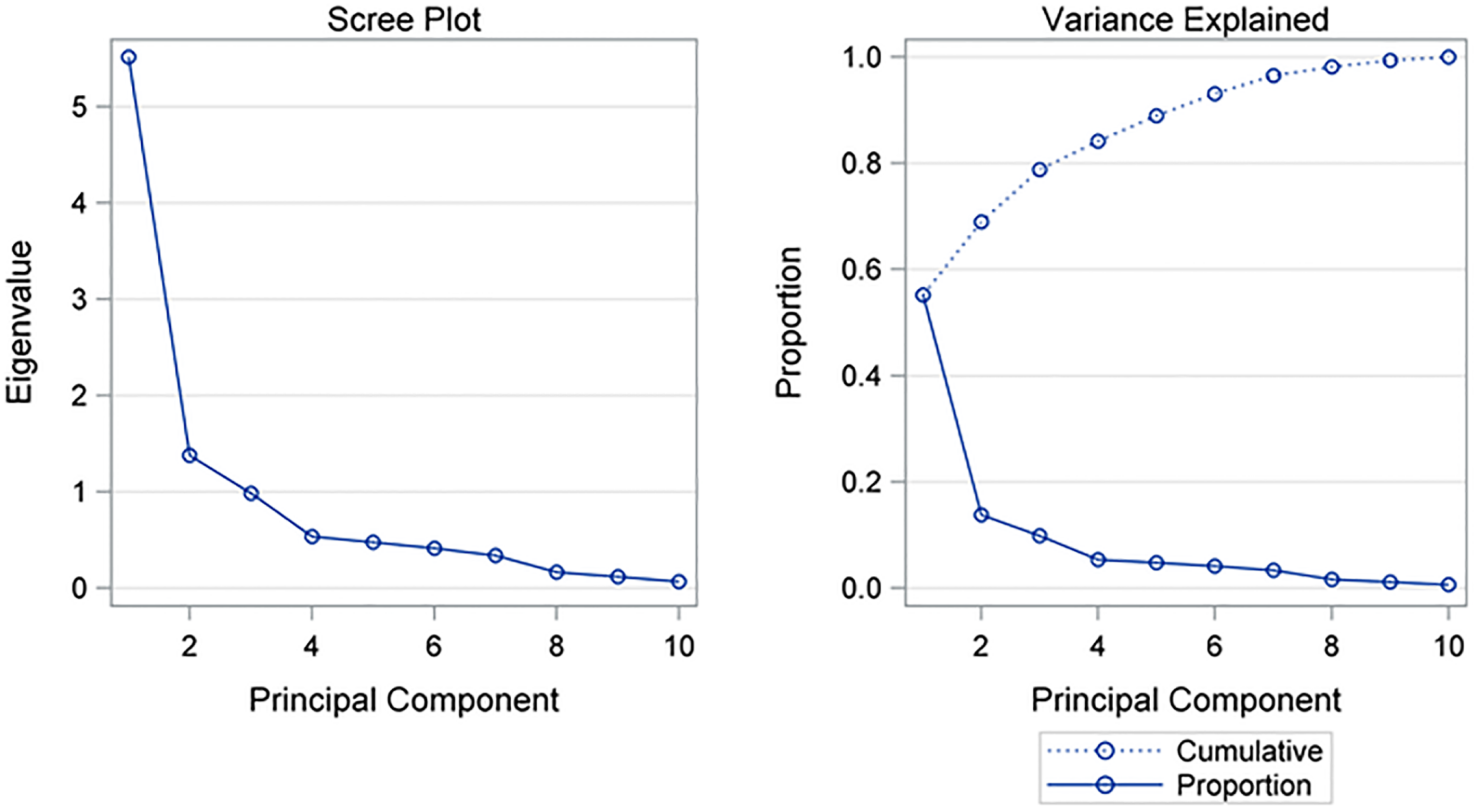
Scree plot for the eigenvalues of the correlation matrix.

Kaiser criterion and the scree plot based on the eigenvalues of the correlation matrix (see Fig 3) indicate that two principal components should be retained. Globally, they account for about 70% of the total variance and individually for 55.16% and 13.79%, respectively.

**Fig 4:**
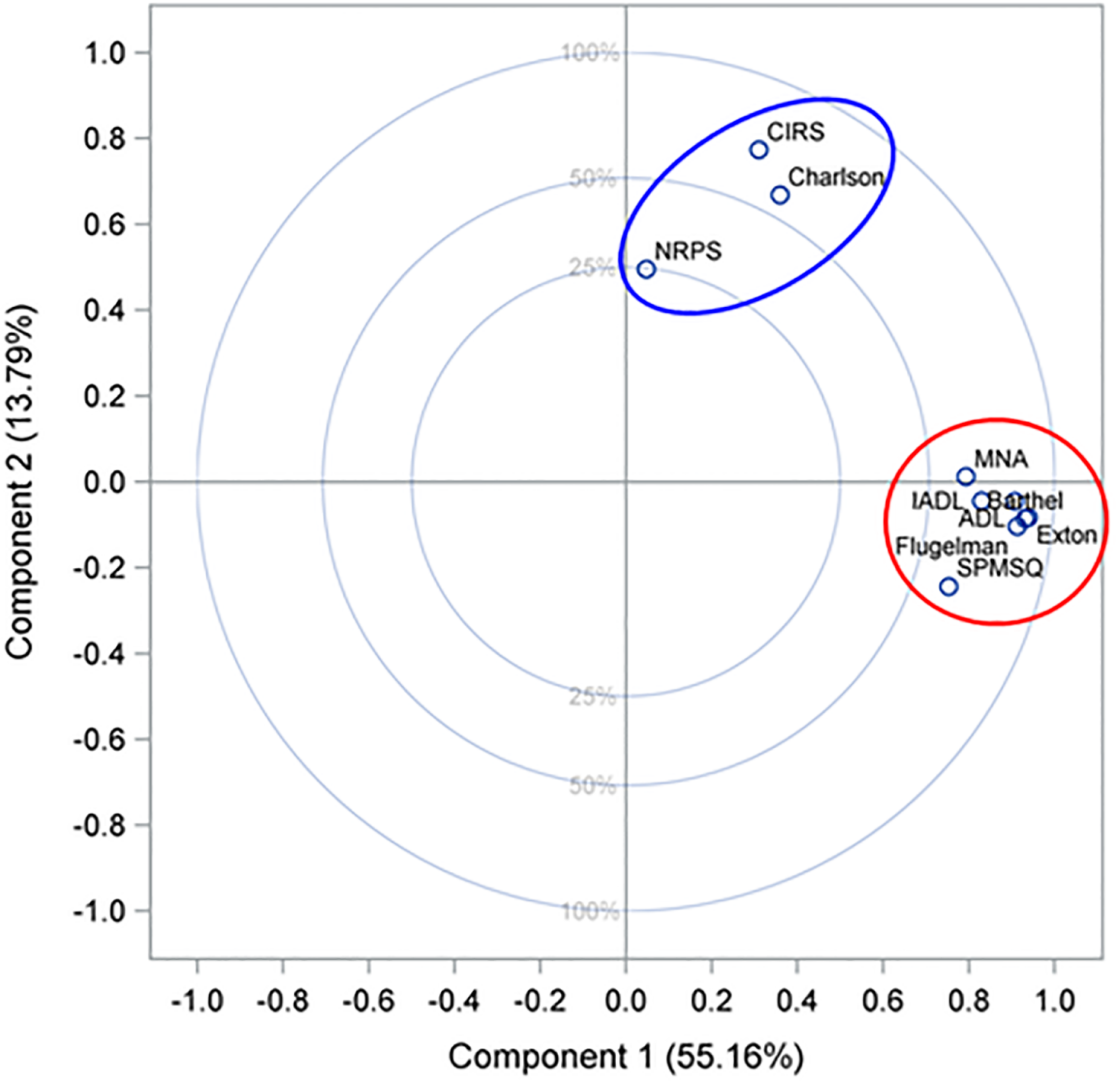
Scores of the questionnaires for the two principal components. Twenty-five percent, 50%, and 100% variance circles are displayed in the plot.

The scores based on standardized data for the two retained principal components were used to plot the questionnaires as shown in Fig 4. Consistently with the preliminary results of cluster analysis, two distinct clusters can be identified visually. The first includes seven questionnaires whose common denominator is their prevalent association with the domains of dependence and frailty. The second cluster consists of the remaining three questionnaires, which share an association with comorbidity. Very similar results were obtained through the sensitivity analysis performed using the information collected at discharge from hospital. In addition, since the eigenvectors’ elements correspond to the weight given to each questionnaire in the calculation of principal component scores, the questionnaires belonging to the two distinct clusters may also be ranked by importance based on the eigenvectors given in Table 2.

**Table 2:** Eigenvectors for the two principal components.

Questionnaire	Principal Component 1	Principal Component 2
**Exton-Smith**	0.399465	*-0*.*070244*
**Barthel Index**	0.396565	*-0*.*072844*
**Flugelman**	0.388654	*-0*.*088245*
**ADL**	0.386328	*-0*.*038803*
**IADL**	0.353256	*-0*.*037438*
**MNA**	0.337868	*0*.*010816*
**SPMSQ**	0.320524	*-0*.*207837*
**CIRS**	*0*.*131917*	0.658778
**Charlson**	*0*.*152875*	0.569000
**NRPS**	*0*.*020224*	0.421903

Following the results of Principal Component Analysis, we can assume that complexity has to be interpreted as a two-dimensional clinical phenomenon, quantifiable through two distinct scores. The first score should measure the first dimension of complexity, which corresponds to the degree of dependence plus frailty as indicated by the first principal component; the second score should be applicable to the second dimension of complexity, which corresponds to the degree of comorbidity as indicated by the second principal component.

#### Concurrent validity

The concept of validity known as “concurrent validity” implies that a variable or measurement is valid if its values are close to the true values of what the variable or measure represents. On this basis, we can assert that PCA conclusions can be considered valid if strong analogies exist between the identified principal components and the tools already validated and used for the same purpose. In this regard, it is worth noting that the principal component 1 and the validated MPI both relate to the domain of dependence plus frailty.

**Fig 5:**
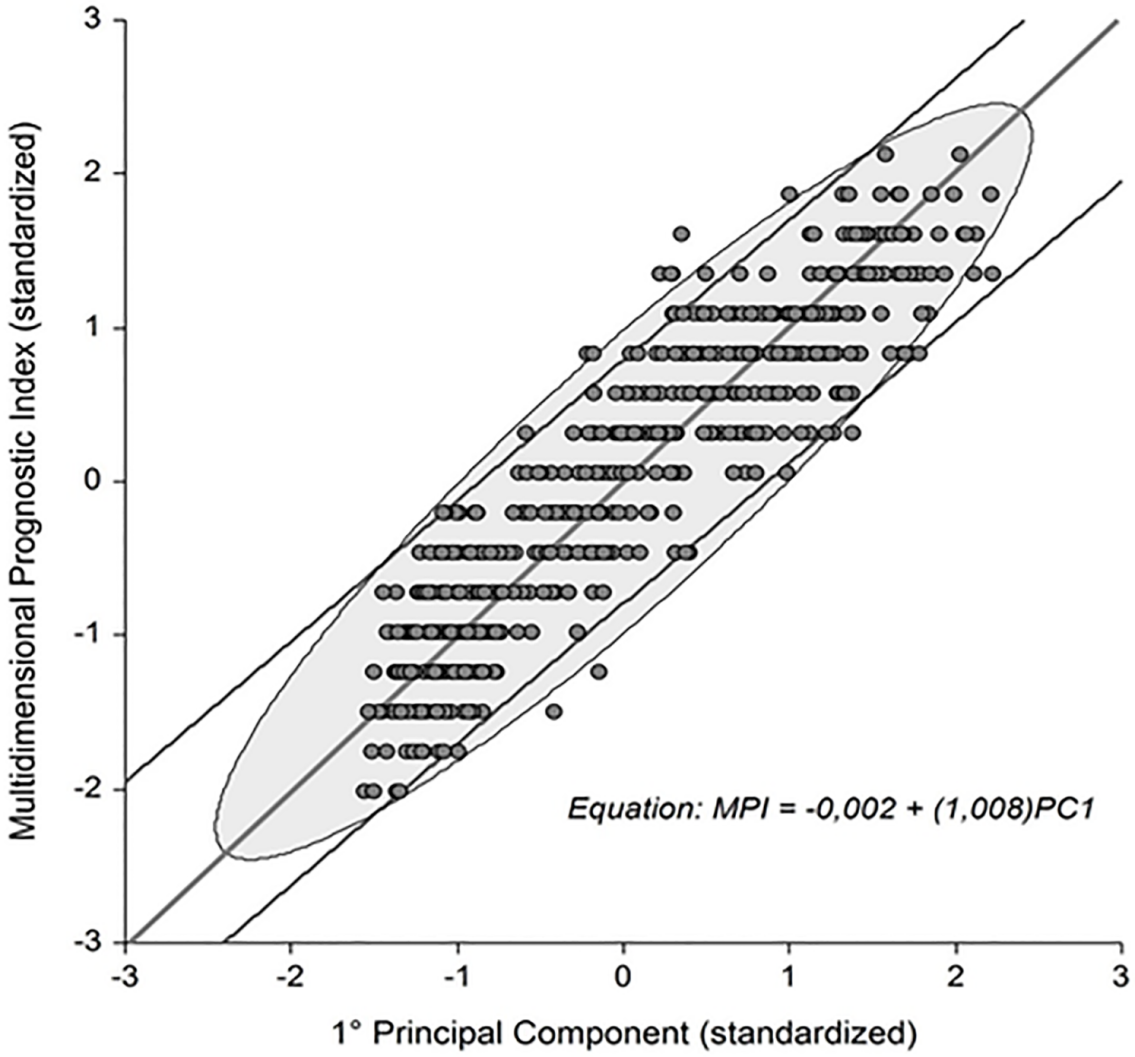
Relationship between principal component 1 and the Multidimensional Prognostic Index (MPI). Regression line, 95% prediction limits, 95% probability ellipse and residuals are displayed in the plot.

Results of the linear regression analysis illustrated in Fig 5 are supportive of a strong agreement and interdependence between the two measurements as confirmed also by the high values of the Kendall’s Tau Correlation Coefficient (≈ 0.77) and of the Lin’s Concordance Correlation Coefficient (≈ 0.92).

### Scores of complexity

#### Definition

The first principal component depends mostly on seven questionnaires which, however, are not designed to provide, when taken individually, an acceptable measure of the degree of dependence plus frailty. Since the first seven elements of the first eigenvector are very similar (see Table 2), one solution would be to approximate the first score of complexity, namely “Complimed Score 1”, to an average, considering the scores of two or more questionnaires related to dependence/frailty.

Based on its correlation with the first principal component score, we found that the best result for the “Complimed Score 1” is a composite index obtained as an average between the Barthel Index score and the Exton Smith score appropriately recalibrated so that they range from 0 (best) to 100 (worst). The R^2^ coefficient was equal to 92.5% indicating that with such a composite score we lose less than 8% of the total information contained in the first principal component. Given that the benefit (simplification, significant reduction of time needed) far exceeds the cost (loss of 8% of information) such an approximation appears more than justified. The formula for calculating “Complimed Score 1” is: **{[100–BI]/100 + [(20-ES)/(20–5)]}x50** with BI and ES indicating Barthel Index and Exton-Smith respectively.

The second principal component depends on three questionnaires with two of them (CIRS and Charlson) precisely designed to provide an indicator of comorbidity. It is therefore reasonable to approximate the second score of complexity, namely “Complimed Score 2”, to one of the two aforementioned questionnaires. Although the weight of CIRS is slightly higher (see Table 2), we decided to focus on the Charlson score because it is easier to obtain, and since studies of the association between comorbidity and mortality found a greater predictive value for the Charlson index when compared with other tools [23]. These two conditions are both advisable for a profitable use of Complimed Scores in clinical practice. The formula for calculating “Complimed Score 2” is: **{min[(CS/14); 1]}x100** with CS indicating Charlson score and 14 the maximum “observed” value for the Charlson score.

#### Clinical applications

It is important to underline that our findings, which clearly indicate a multidimensional nature of complexity, hinder a straightforward scoring of patient’s global complexity. In fact, we can measure the individual components of complexity, namely dependence/frailty and comorbidity, but cannot summarize the two measurements through pre-defined categories of complexity (e.g. mild, moderate and severe). However, this issue can be resolved by using a direct manifestation of complexity—for instance mortality, as dependent variable in a risk function with the identified domains of complexity (dependence/frailty and comorbidity) as independent predictors. In this way, mortality would act as substitute for complexity and the combined effects of Complimed scores on death would indirectly provide an overall measurement of complexity. The definition and implementation of risk functions based on Complimed scores should be the subject of further studies.

## Discussion

In our study, we have shown that the complexity of hospitalized patients may be described as a two-dimensional phenomenon (functional dependence/frailty + comorbidity), and we have been able to define a new comprehensive tool for scoring complexity. Additionally, the FADOI-Complimed Scores need only three questionnaires to be filled out (Barthel, Exton-Smith, Charlson), with a total of 34 items—around half of that required by the MPI index (63 items). This should make our tool easier to use.

Patients hospitalized on medical wards are generally older and suffer from multiple concomitant diseases. Moreover, they are heterogeneous in terms of illness severity, risk of adverse events, functional and cognitive status, personal priorities and preferences, and poor treatment outcomes [3, 24]–in other words, “complex”. However, what makes patients complex and how to measure their complexity are still unresolved questions (6), and despite the availability of several tools for a multidimensional approach and prognosis, complexity is not routinely assessed in clinical practice. Therefore, our aim was to develop a new tool that would provide a valid and comprehensive definition of complexity, but that was also practical enough to enable its systematic use. We did this by considering a wide number of domains and by using a robust statistical approach for their analysis.

Multimorbidity is one of the strongest predictors of mortality in different patient settings [25, 26], and it is not surprising that this significantly contributes to the definition of complexity in our model. In this perspective, our study seems to confirm that there is an only partial overlap between the concepts of complexity and frailty, since the latter is one of the two major axes that define the phenomenon of complexity, but the domain of comorbidity/clinical status has a relevant role as well. However, it is well known that the type and severity of illnesses, as well as the degree to which the diseases interact are important prognostic determinants. Moreover, a multitude of patient-level factors independent of specific comorbid conditions may complicate clinical management and affect outcomes. From this point of view, and as previously suggested [1, 27], our finding that comorbidity is a significant—although not the most important—component of complexity seems plausible.

The multidimensional concept of patients’ complexity, involving interactions between biological, socioeconomic, cultural, environmental and behavioral forces as health determinants is a reasonable approach and it has already been proposed (2). However, in order for complexity to be assessable in routine clinical practice, the identification of those components/domains which mostly contribute to the definition of patients’ conditions and correlate to clinical outcomes is required. According to our results, the questionnaires related to functional dependence/frailty were identified as those that principally described the complexity of hospitalized medical patients. This finding is coherent with the opinion of authors who suggested that changes in a patient’s health status with an impairment of functional performance due to failure of the homeostatic reserves to pathological stressors (i.e. “frailty”), may be an important component of complexity and determinant of outcome [28].

One critical point related to the reliability and consistency of our results is the choice of domains and specific information we explored in our study, to find a synthetic but representative definition of complexity. We chose to refer to thirteen well-known and already validated questionnaires, and this should support our assessment. Nevertheless, a potential limitation of our analysis is the assumption that the panel of thirteen surveyed questionnaires contains all the information related to complexity. We cannot exclude the possibility that some relevant variables have been left out; however, we believe it is quite unlikely that such an eventuality may have altered the major study conclusions.

Another potential drawback is the “a priori” exclusion of unclustered questionnaires from the main analysis, having assumed that they are not sufficiently relevant in terms of complexity. In fact, we cannot rule out the possibility that complexity could have been expressed in a more articulated form by including, alongside the two main domains of dependence/frailty and comorbidity, even three minor axes corresponding to clinical stability (MEWS), depression (GDS) and adherence to therapy (Morisky). However, it is unlikely that the contribution of these three minor axes to the total amount of complexity would be so important as to warrant their inclusion in the model, if balanced with the additional information to be collected and the interpretability issues that would arise. Further, the hypothesis that complexity could be a clinical phenomenon with five domains instead of two contrasts with the *Occam’s razor* principle which states that the simpler explanation is usually the most accurate. Finally, we would stress that the three unclustered questionnaires are to be tested anyway as additional prognostic factors in mortality risk functions together with the Complimed scores: this would for all practical purposes reduce the need to determine whether they actually contribute or not to complexity as further independent domains.

## Conclusions

In conclusion, the general criteria for the use of an index in routine clinical practice are that it should be valid and comprehensive, simple to record and calculate, its component items clinically important in their own right, and it should be predictive of a major measurement of disease severity (e.g. mortality, future exacerbations, healthcare provision) [29]. The FADOI-Complimed Score(s) is a new tool that may be useful for the routine evaluation of complexity in patients hospitalized in medical settings, having been developed to be equally if not more comprehensive than available ones, as well as simple to use. The two clinically relevant domains of functional dependence/frailty and comorbidity appear as the main determinants of complexity in these patients, and they may be assessed by collecting information included in three universally used questionnaires, which take around 10 minutes. Further analyses should evaluate the prognostic value of this tool, and therefore its potential role in clinical practice.
